# Exploratory investigation of virtual lesions in gastrointestinal endoscopy using a novel phase‐shift method for three‐dimensional shape measurement

**DOI:** 10.1002/deo2.381

**Published:** 2024-05-08

**Authors:** Taku Sakamoto, Ichiro Oda, Takuma Okamura, Hourin Cho, Naoya Toyoshima, Satoru Nonaka, Haruhisa Suzuki, Tatsuya Nakamura, Daichi Watanabe, Keigo Matsuo, Kazunari Hanano, Tetsuhide Takeyama, Shigetaka Yoshinaga, Yutaka Saito

**Affiliations:** ^1^ Endoscopy Division National Cancer Center Hospital Tokyo Japan; ^2^ Division of Science and Technology for Endoscopy Exploratory Oncology Research and Clinical Trial Center National Cancer Center Tokyo Japan; ^3^ Optical Engineering Olympus Medical Systems Corporation Tokyo Japan

**Keywords:** dimensional measurement accuracy, endoscopes, gastrointestinal neoplasms, gastrointestinal tract, three‐dimensional imaging

## Abstract

Accurate measurement of the size of lesions or distances between any two points during endoscopic examination of the gastrointestinal tract is difficult owing to the fisheye lens used in endoscopy. To overcome this issue, we developed a phase‐shift method to measure three‐dimensional (3D) data on a curved surface, which we present herein. Our system allows the creation of 3D shapes on a curved surface by the phase‐shift method using a stripe pattern projected from a small projecting device to an object. For evaluation, 88 measurement points were inserted in porcine stomach tissue, attached to a half‐pipe jig, with an inner radius of 21 mm. The accuracy and precision of the measurement data for our shape measurement system were compared with the data obtained using an Olympus STM6 measurement microscope. The accuracy of the path length of a simulated protruded lesion was evaluated using a plaster model of the curved stomach and graph paper. The difference in height measures between the measurement microscope and measurement system data was 0.24 mm for the 88 measurement points on the curved surface of the porcine stomach. The error in the path length measurement for a lesion on an underlying curved surface was <1% for a 10‐mm lesion. The software was developed for the automated calculation of the major and minor diameters of each lesion. The accuracy of our measurement system could improve the accuracy of determining the size of lesions, whether protruded or depressed, regardless of the curvature of the underlying surface.

## INTRODUCTION

Accurate measurement of the size of a lesion, determined as the length between two points, is important during the endoscopic examination to inform the clinical management of gastrointestinal neoplasms. For example, differentiation between a lesion 20 mm in size and one 30 mm in size can alter the indication for endoscopic treatment of early gastric cancer.^1^ Similarly, the distance between the cardia of the stomach and the oral side of a tumor determines the indication for proceeding with stomach‐preservation procedures.^2^ Measurement accuracy is also crucial to inform appropriate treatment for gastrointestinal neoplasms, as treatment selection can have a determinant effect on patients’ cancer prognosis and quality of life postoperatively. Furthermore, lesions that exhibit distinct elevations and depressions can be clearly identified by radiological examinations (such as gastric computed tomography colonography, or fluoroscopy), whereas lesions, where only subtle changes in color tones or mucosal patterns form the boundary, are better evaluated by endoscopic examination. However, for measuring the size of lesions or distances from certain reference points, radiographic imaging offers higher objectivity, whereas endoscopy can make accurate and objective assessment difficult because of various factors. The accuracy of the length measurement of these lesions can also be influenced by the curvature of the gastric surface, which can lead to subjective errors and poor oncological treatment selection. As such, an objective measurement method for lesions on the curved gastric surface would be desirable to inform standardized management of gastrointestinal neoplasms. Forceps measures that are widely used in clinical settings are also unsuitable because they can only measure straight distances. To address this issue, we developed a three‐dimensional (3D) visual inspection system, using a phase‐shift method, to improve the measurement accuracy of irregular surfaces, such as indentations and swellings, that are difficult to predict on two‐dimensional (2D) images. Herein, we report the measurement accuracy of this novel system. The phase‐shift method is one of the primary shape measurement methods that projects a stripe pattern onto an object and shifts the pattern position sequentially to obtain shape data of the object from the pattern distortion information.

## TECHNIQUE

### Statement of ethics

This study was approved by the National Cancer Centre Hospital Institutional Review Board on January 5, 2019 (2018‐250).

### Development of the 3D shape measurement system

**FIGURE 1:**
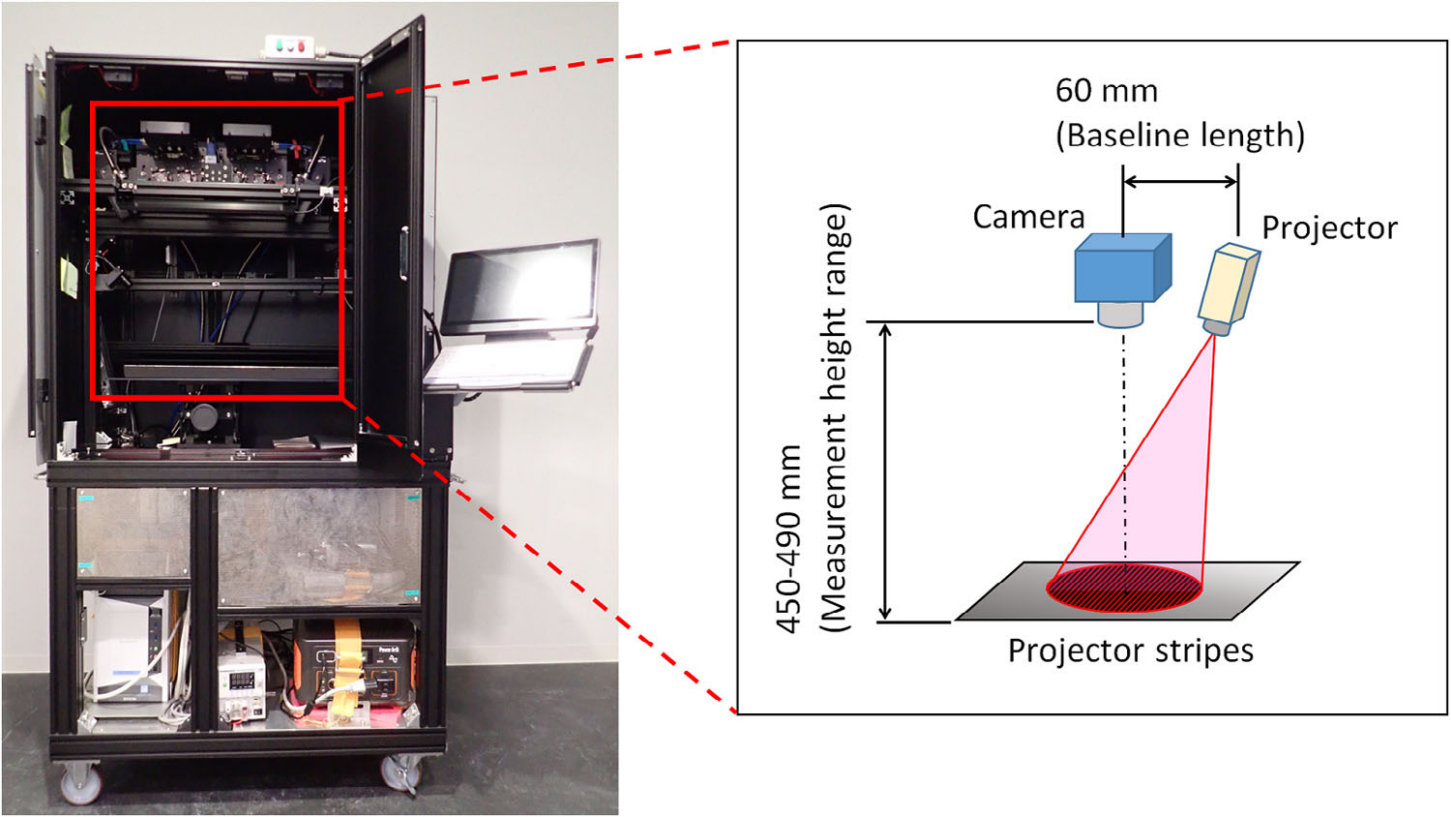
A representative picture of the desktop system for our three‐dimensional (3D) shape measurement system, showing the equipment used and the layout of the optical system.

Our 3D shape measurement system (MS) includes a miniature projector, developed specifically for this study by the Japan Telegraph and Telephone Corporation, as well as an industrial camera (DFK33UX264) and lens (MVL50TM23). The distance from the camera to a target surface is set to a standard height of 470 mm, with a field of vision of 79 mm (width, W) × 66 mm (height, H) at the standard height. The projector outputs a red stripe pattern to scan the target surface, with approximately 15 stripes included in the field of vision at the standard height on the target surface. The horizontal (baseline) length between the camera and the projector is 60 mm, with a 450–490 mm range of measurement available at the standard height of the camera from the target surface (Figure 1). The projector and camera are integrated into a desktop system. Displacement of the projected red stripe pattern for scanning of the target surface relative to the configuration of the projector and camera allows for a high‐definition derivation of the 3D geometric shape of the scanned object.^3^ Of note, we confirmed that the camera and projector system can be mounted on an endoscope while the system projector and camera were integrated into a desktop system in this study.

### Height measurement accuracy using a porcine stomach model

**FIGURE 2:**
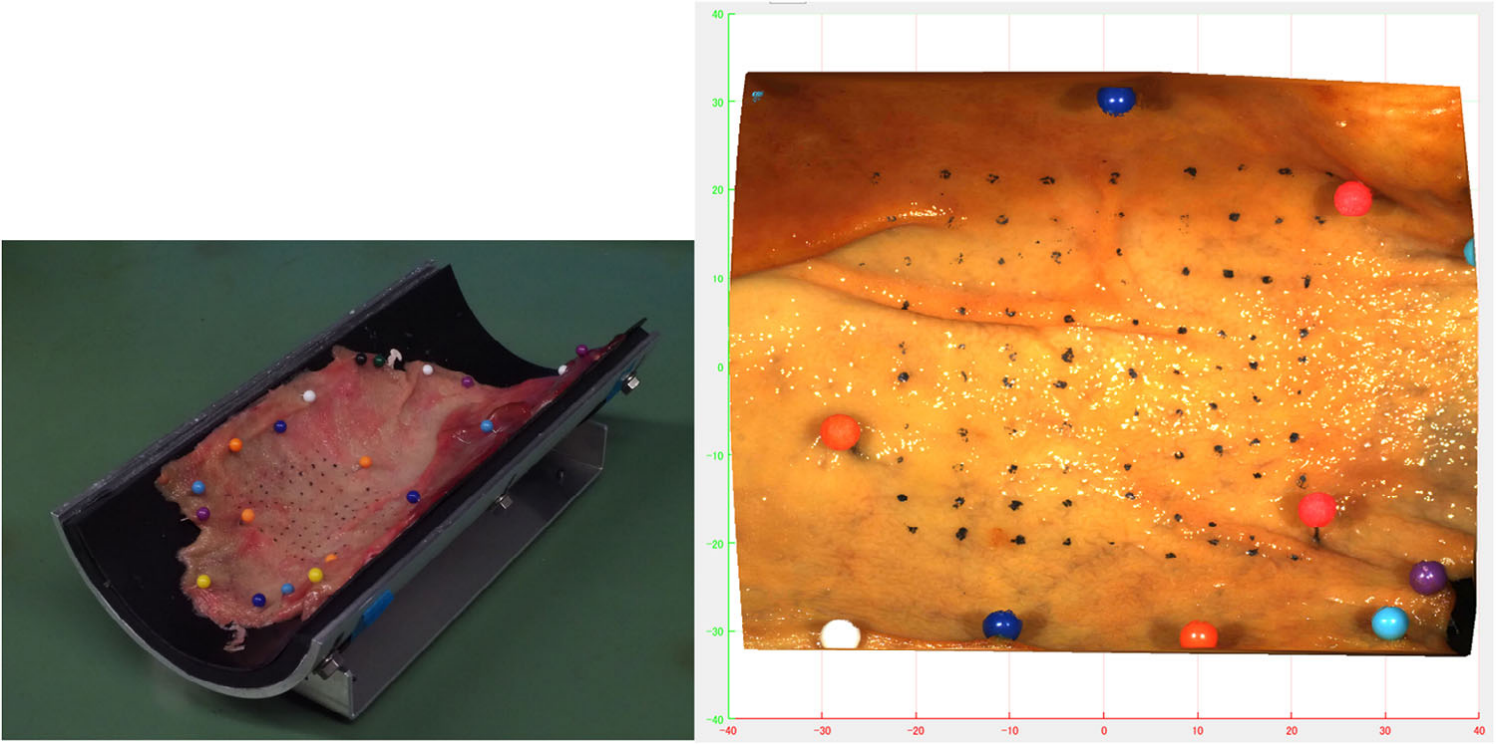
Porcine stomach tissue, attached to the half pipe jig, with an inner radius of 21 mm and 88 measurement points for height measurement.

We prepared a specimen comprising a porcine stomach attached to a half‐pipe jig, with an inner radius of 21 mm, consistent with the curvature of a 3D model of the human stomach, to evaluate the accuracy of our MS (Figure 2). For positional alignment, three alignment marks were added to the specimen using graphite, and 88 measurement points were added for measurement evaluation. The reason for using graphite was to precisely focus the measurement microscope (MM). Height data were calculated as the average value of the height on the surface of the tissue at two points (located at ±1.5 mm from each measurement point) since the graphite placed at the measurement points is not a tissue component.

The MM is a special microscope with a measurement function that comprises a microscope, a stage moving in 3 axes, and a monitor for displaying measured values. We used the MM as reference equipment to evaluate the accuracy of the MS, but it was not used at the actual clinical site. Measurement data obtained from our shape MS (MS data) were compared with the MM data as a reference with correction of the position of measurement points using the alignment markings. The MM data include the X‐, Y‐, and Z‐coordinates of the 88 measurement points set at a grid pitch of approximately 5 mm within a vertical range of 50 mm and a horizontal range of 45 mm of the porcine stomach. The accuracy and precision of the MS data were evaluated for 2640 data points (30 repeat measurements of the 88 measurement points).

## RESULTS

**FIGURE 3:**
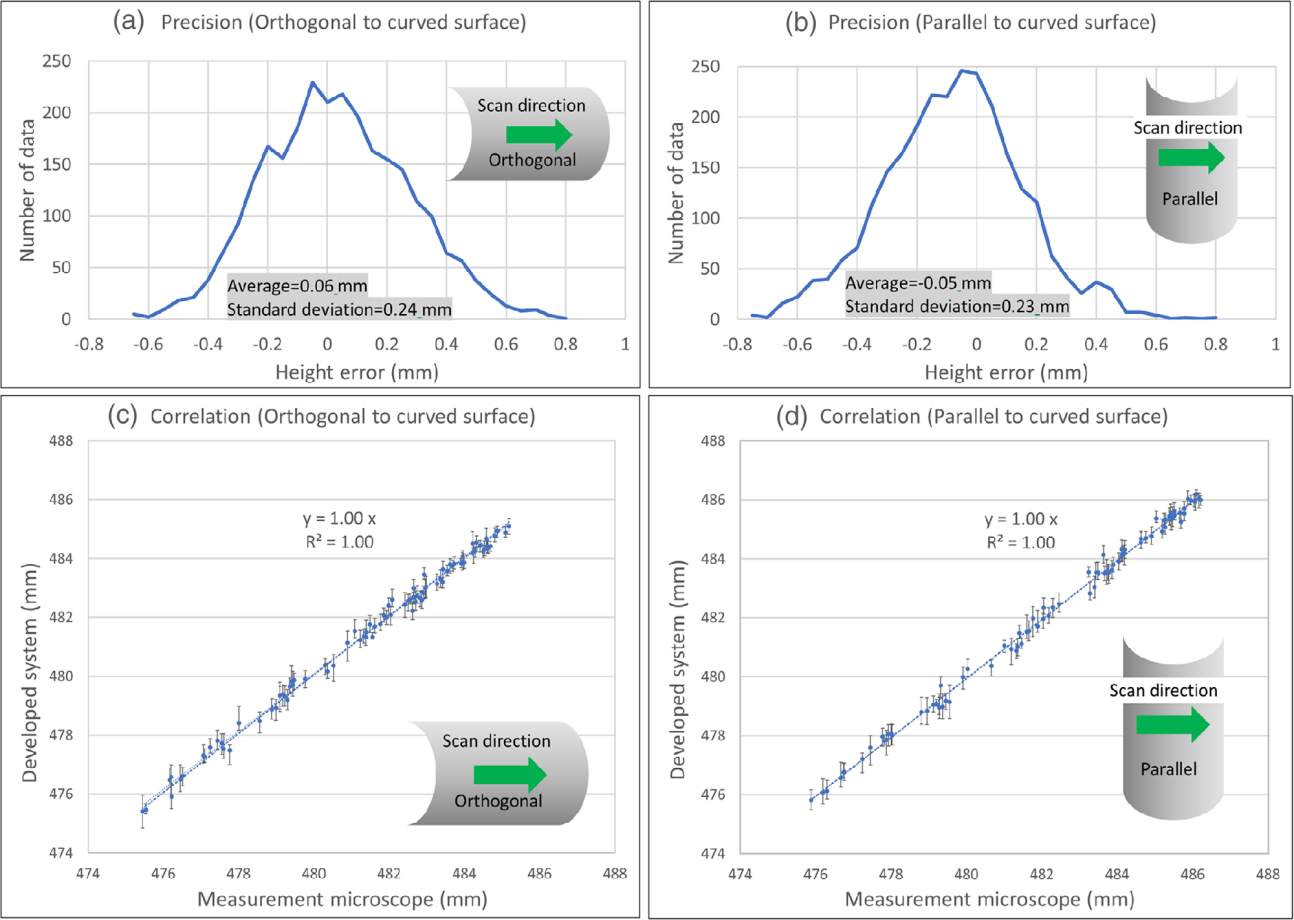
Histogram of the error in height measurement for the two directions of scanning. Height error (*x*‐axis) and number of data (*y*‐axis) (a) from an orthogonal to a curved surface and (b) from a parallel to a curved surface. σ, standard deviation. Correlation between our 3D shape measurement system (*y*‐axis) and the reference measurement microscope data (*x*‐axis) for the two directions of scanning (c) from an orthogonal to a curved surface and (d) from a parallel to a curved surface.

The standard deviation of the difference in height measurements between the MS and MM data was 0.24 mm (3 standard deviation cut‐off, 0.72 mm) over the 2640 data points included in the analysis (Figure 3). The accuracy of measurements was evaluated as the correlation between the MM and MS data (Figure 3). Both data sets were highly correlated, with an R^2^ value of 0.99 for scan directions from an orthogonal to a curved surface and from a parallel to a curved surface.

**FIGURE 4:**
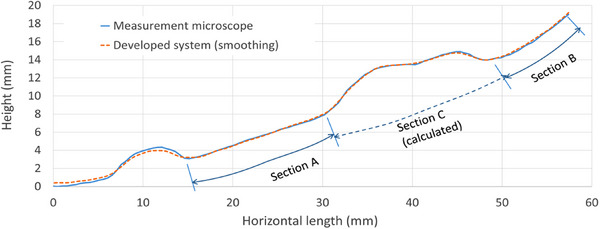
Cross‐sectional profile of the protruded lesion in the plaster model of the stomach, with height (*y*‐axis) and length (*x*‐axis) measures of both the measurement microscope (blue line) and our visual system (red line). Path length for sections A and B, including the difference between measurement microscope and measurement system measures.

The accuracy of measurement in the X‐Y direction was evaluated by comparing the MS data with the graph paper as a reference, with the difference in measurement being <0.15 mm. As an additional test, the accuracy of the length measurement was evaluated for three sections (A, B, and C) of the protruded lesion in the plaster stomach model (Figure 4), with the height and length measures for the MM (blue line) and MS (red line) shown, with a smoothing filter applied to the MS data. The MM and MS measures, with and without smoothing filtering, are shown in Table S1. The difference between the MM and MS length measures for sections A and B was 0.37 and 0.55, respectively, under normal conditions. Applying the smoothing filter improved the accuracy by 0.03 and 0.0 mm in the MM and MS, respectively. Therefore, the error in the length measurement on the curved surface using the MS was <5%. Furthermore, the length of section C was estimated by creating a virtual inferior surface and using polynomial approximation, with reference to sections A and B. The length of section C was estimated to be 20.6 mm.

**FIGURE 5:**
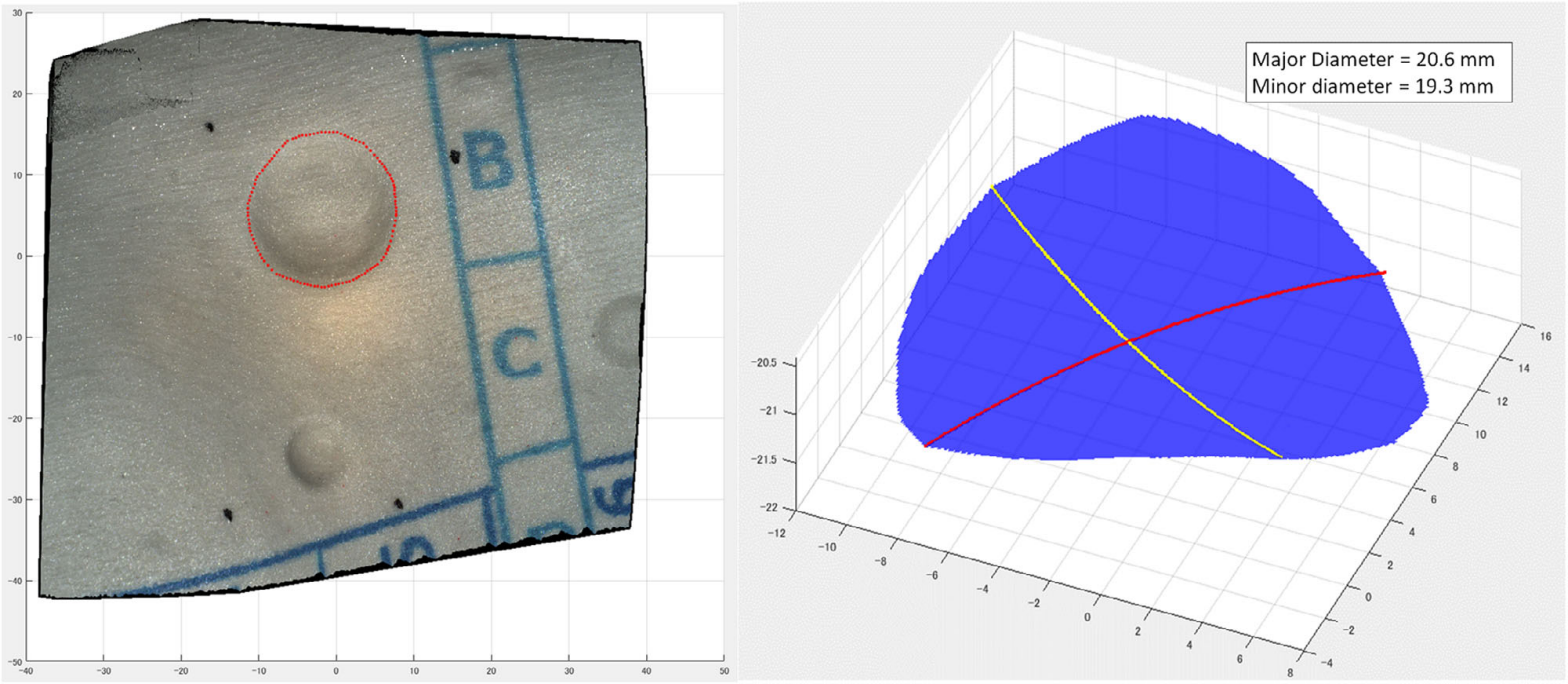
Shape analysis software, with a path length approximation function, is used to calculate the major and minor diameters of a protruded lesion on a curved surface (left panel). The re‐created lesion is shown in the right panel.

Shape analysis software, equipped with a path length approximation function, was used to calculate the major and minor diameters of the lesion (Figure 5). With the region of interest (ROI) set to surround the lesion, a virtual surface was created from which the extracted lesion could be set on the curved surface, with the major and minor diameters automatically calculated.

## DISCUSSION

Current indications for endoscopic resection of gastrointestinal tumors are largely based on the estimated size of the tumor and depth of invasion, which are indicative of the oncological grade.^2^ However, predicting the size of the lesion based on images obtained only using the cameras mounted on endoscopes can lead to subjective variability owing to the barrel distortion characteristic of the fisheye lens of endoscopic cameras.^4^, ^5^, ^6^ This variability is further influenced by the curvature of the gastric surface, local deformation of the tissue (such as stretching), and the presence of air caused by insufflation during surgery. Our novel MS addresses this issue by providing 3D measurement data on the curved surface. Our MS‐based path length measures, using 3D data within an ROI, had sufficient accuracy and precision (Figure 3) for measuring the path length, regardless of the curvature of the underlying surface, to be applied to an actual lesion (File S1). This accuracy in the measurement of the length of protruded lesions is a distinct advantage of MS in informing clinicians on the optimal endoscopic treatment for gastric lesions. We achieved a higher accuracy of path length measures of protruded lesions than that of currently used linear measures by extracting the lesion and applying a path length approximation function to calculate the distance along the curvature of the virtual surface (Figures 4 and 5). As such, our system could be useful in determining the size of early gastrointestinal cancer, which is a determinant parameter for oncological treatment selection.^7^, ^8^ Although further comparison of the 3D MS data to histological information is warranted, we expect that the MS measures will be as accurate for in vivo lesions on a curved surface in practice as for the experimental lesions in our porcine stomach model. Our system using low‐pass filtering should also shorten the endoscopic measurement time for in situ use during endoscopy in humans, which is currently several tens of seconds owing to the effects of organ motion and natural physiological tremor of the endoscopist. We are also currently aiming to improve the general versatility of our system to eliminate the need for a dedicated endoscope and to ensure accuracy for different approaches to the target lesion. As our technology can be mounted on a small surface, it has the potential to be incorporated into current endoscope products and probe types.

If we compare the proposed techniques with past size measurement technologies, Fuji Film's Virtual Scale Endoscope using laser light was able to improve the estimation accuracy of polyp size by up to 84%.^9^ However, a drawback of this system is that the endoscope's optical axis and the plane of the lesion need to be perpendicular during measurement, as deviation from this condition leads to decreased estimation accuracy. Conversely, in a study by Sudarevic et al., the authors proposed estimating the diameter of a water jet near a polyp and comparing it with a bounding box to approximate polyp size, achieving a median error of 7.4%.^10^ Nevertheless, controlling the position of the polyp and the water jet is necessary to maintain accuracy. Further, these techniques rely on 2D size estimation, making it impossible to measure depth along curved surfaces. Additionally, EndoAngel employs AI‐based polyp size measurement with a relative depth estimation error of 11.3% and a measurement accuracy of 89.9%, but as this feature is not implemented in an endoscope, direct comparison is challenging.^11^ However, while EndoAngel's measurement principle is a black box, the proposed phase‐shift method has a clear measurement principle, eliminating uncertain factors. Further, our proposed system does not restrict the endoscope's position but requires a separate device for projecting stripes.

The present study has some limitations. First, the experiment was conducted on a model and not on a human body. Although there were no safety concerns during the experiment, it is essential to verify these findings in humans for practical application, as milestones are steadily surpassed. Second, the milestones for practical use can only be achieved through verification in human subjects. Third, it is imperative to reduce the size of optical devices and incorporate them into endoscopes, followed by thorough verification.

In conclusion, we have developed a novel shape MS to obtain 3D measurements of lesions of different shapes (whether protruded or depressed), regardless of the curvature of the underlying surface. Therefore, our methods are appropriate for measuring lesions in the gastrointestinal tract. The next step will be to evaluate the safety and accuracy of our system in vivo.

## CONFLICT OF INTEREST STATEMENT

The research reported in this paper was conducted as part of a collaborative effort between Olympus Medical Systems Corporations and National Cancer Center Hospital, Tokyo. Olympus Medical Systems Corporations provided resources and expertise for the research.

## Supporting information


**FIGURE S1** The 3‐dimensional model of the flat elevated lesion.

**FIGURE S2** Process of automatic distance measurement on the 3‐dimensional model.

**TABLE S1** Comparison of the path length with the actual measurements.

**FILE S1 **Development of a three‐dimensional shape measurement system using a phase‐shift method.
