# Supplementary figures and images for: Exploratory investigation of virtual lesions in gastrointestinal endoscopy using a novel phase‐shift method for three‐dimensional shape measurement
Source: DEN Open. 2024 May 8;5(1):e381. doi: 10.1002/deo2.381 (PMC11079539; doi:10.1002/deo2.381)

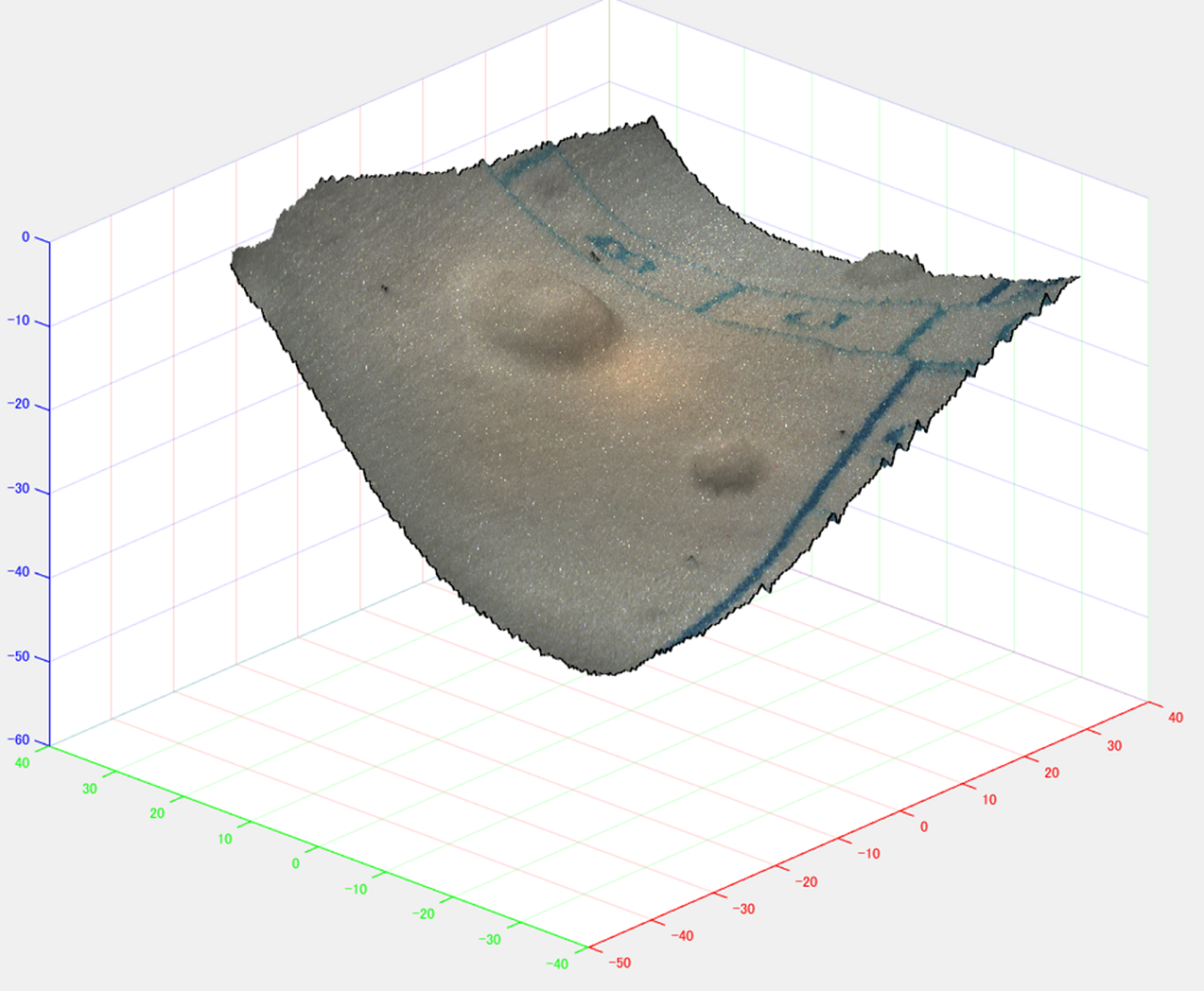

Supplement: Supplementary file 1 — FIGURE S1 The 3‐dimensional model of the flat elevated lesion. [file DEO2-5-e381-s002.tif]

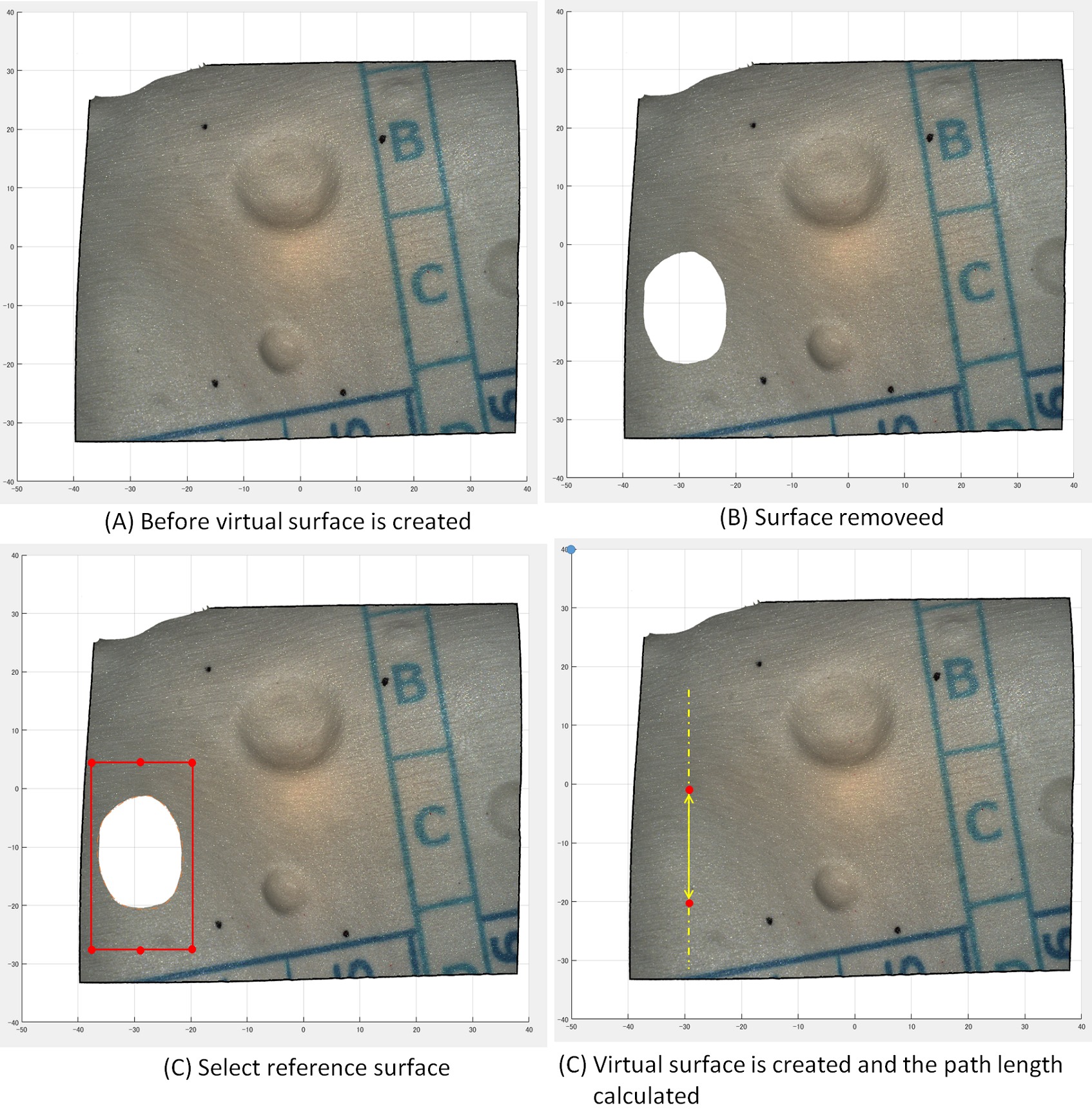

Supplement: Supplementary file 2 — FIGURE S2 Process of automatic distance measurement on the 3‐dimensional model. [file DEO2-5-e381-s001.tif]
