# Supplementary material for: Exploratory investigation of virtual lesions in gastrointestinal endoscopy using a novel phase‐shift method for three‐dimensional shape measurement
Source: DEN Open. 2024 May 8;5(1):e381. doi: 10.1002/deo2.381 (PMC11079539; doi:10.1002/deo2.381)
Supplement: Supplementary file 3 — TABLE S1 Comparison of the path length with the actual measurements. [file DEO2-5-e381-s003.docx]

Supplementary table 1. Comparison of the path length with the actual measurements

| Method | Path length (mm) | Error from the measured value | Error rate against the measured value |
| --- | --- | --- | --- |
| Measures of the microscope | 21.11 mm | 0 mm | 0 % |
| Calculated value on the original surface | 21.03 mm | -0.09 mm | -0.40 % |
| Calculated value on the virtual surface | 21.02 mm | -0.10 mm | -0.45 % |
