# Supplementary material for: Exploratory investigation of virtual lesions in gastrointestinal endoscopy using a novel phase‐shift method for three‐dimensional shape measurement
Source: DEN Open. 2024 May 8;5(1):e381. doi: 10.1002/deo2.381 (PMC11079539; doi:10.1002/deo2.381)
Supplement: Supplementary file 4 — FILE S1 Development of a three‐dimensional shape measurement system using a phase‐shift method. [file DEO2-5-e381-s004.docx]

**Supplementary document 1: Development of a three-dimensional shape measurement system using a phase-shift method**

*A simple accuracy check of the path length on a virtual curved surface*

We checked the path length on a virtual curved surface to evaluate the accuracy of the calculation of the major or minor diameters of a protruded lesion.

The method using the 3-dimensional model shown in Supplementary figure 1 was as follows:

1. We generated a virtual surface on a curved surface of the model with a radius of curvature of approximately 40 mm (Supplementary figure 2 A-C).
2. We extracted the path length (Supplementary figure 2 D) before and after the virtual surface was generated.
3. We calculated the error, which was the difference between the path length as above 1) - 2), and the error in the distance was measured using an optical microscope with the measurement function.

*RESULTS*

The cross-sectional profile extracted from the original surface or virtual surface is shown in Figure 3. In the figure, the cross-sectional profile measured with the microscope is shifted downward by 1 mm. The path lengths obtained by integrating the minute interval distance are presented in Figure 4.

Overall, the error rate of measurement is <0.5% in conditions where the path length is approximately 20 mm on the surface with a radius of curvature of approximately 40 mm.
